# NAD^+^ improves cognitive function and reduces neuroinflammation by ameliorating mitochondrial damage and decreasing ROS production in chronic cerebral hypoperfusion models through Sirt1/PGC-1α pathway

**DOI:** 10.1186/s12974-021-02250-8

**Published:** 2021-09-16

**Authors:** Yao Zhao, Jiawei Zhang, Yaling Zheng, Yaxuan Zhang, Xiao Jie Zhang, Hongmei Wang, Yu Du, Jian Guan, Xiuzhe Wang, Jianliang Fu

**Affiliations:** 1 Department of Neurology, Shanghai Jiao Tong University Affiliated Sixth People’s Hospital, 600 Yishan Road, Shanghai, 200233 China; 2 Shanghai Key Laboratory of Sleep Disordered Breathing, Shanghai Jiao Tong University Affiliated Sixth People’s Hospital, Shanghai, China

**Keywords:** Chronic cerebral hypoperfusion, Microglia, NAD^+^, Mitochondria, ROS

## Abstract

**Background:**

Microglial-mediated neuroinflammation plays an important role in vascular dementia, and modulating neuroinflammation has emerged as a promising treatment target. Nicotinamide adenine dinucleotide (NAD^+^) shows anti-inflammatory and anti-oxidant effects in many neurodegenerative disease models, but its role in the chronic cerebral hypoperfusion (CCH) is still unclear.

**Methods:**

The bilateral common carotid artery occlusion (BCCAO) was performed to establish CCH models in Sprague-Dawley rats. The rats were given daily intraperitoneal injection of NAD^+^ for 8 weeks. The behavioral test and markers for neuronal death and neuroinflammation were analyzed. Mitochondrial damage and ROS production in microglia were also assessed. RNA-seq was performed to investigate the mechanistic pathway changes. For in vitro studies, Sirt1 was overexpressed in BV2 microglial cells to compare with NAD^+^ treatment effects on mitochondrial injury and neuroinflammation.

**Results:**

NAD^+^ administration rescued cognitive deficits and inhibited neuroinflammation by protecting mitochondria and decreasing ROS production in CCH rats. Results of mechanistic pathway analysis indicated that the detrimental effects of CCH might be associated with decreased gene expression of PPAR-γ co-activator1α (PGC-1α) and its upstream transcription factor Sirt1, while NAD^+^ treatment markedly reversed their decrease. In vitro study confirmed that NAD^+^ administration had protective effects on hypoxia-induced neuroinflammation and mitochondrial damage, as well as ROS production in BV2 microglia via Sirt1/PGC-1α pathway. Sirt1 overexpression mimicked the protective effects of NAD^+^ treatment in BV2 microglia.

**Conclusions:**

NAD^+^ ameliorated cognitive impairment and dampened neuroinflammation in CCH models in vivo and in vitro, and these beneficial effects were associated with mitochondrial protection and ROS inhibition via activating Sirt1/PGC-1α pathway.

## Introduction

Vascular dementia (VaD) is the second most common form of dementia [1], and the main pathogenesis of VaD is caused by chronic cerebral hypoperfusion (CCH) [2]. The main pathological changes include increased neuroinflammation and oxidative stress, disturbed mitochondrial function and lipid metabolism, and downregulated expression of growth factors [3]. Among them, neuroinflammation and mitochondrial damage play roles in the etiology of CCH-induced cognitive dysfunction [4].

Microglia are the main inflammatory cells in central nervous system (CNS) [5]. Microglia continuously monitor the brain environment and are activated in response to diverse cues, including both endogenous proteins and externally derived environmental toxins [6]. However, unregulated microglial activation is a contributing mechanism of neuronal damage in neurodegenerative diseases. Mounting evidence indicates that microglial activation contributes to neuronal damage in neurodegenerative diseases [7]. It has been found that the number of microglial cells is increased following cerebral hypoperfusion [8–10], and activated microglia release tumor necrosis factor (TNF)-α, interleukin (IL)-1β and IL-6, and other pro-inflammatory factors that mediate secondary brain damage. These pro-inflammatory factors penetrate into the white matter to cause damage and neuron loss [2], which ultimately lead to learning and memory dysfunction [4, 11]. Targeting microglia has been considered as a promising strategy to treat the cognitive dysfunction and brain damage caused by CCH.

Mitochondria are important organelles that maintain the integrity and normal function of microglia through regulating pathways of cell metabolism and viability [12]. However, recent studies have shown that mitochondria play a vital role in coordinating innate and adaptive immune responses, and thus link neurodegeneration and neuroinflammatory processes [13–15]. Mitochondrial dysfunction and the resulting reactive oxygen species (ROS) have been proposed as pivotal roles in the initial and the continuous activation of microglia [16, 17]. Elevated ROS in microglia causes activation of inflammatory and cell death pathways [18, 19]. Thus, therapeutic methods by targeting ROS in microglia may be helpful to reduce neuroinflammatory damage [20, 21].

Nicotinamide adenine dinucleotide (NAD^+^), also known as coenzyme I, is a key signaling molecule that regulates intermediary metabolism [22]. NAD^+^ acts as a coenzyme in the redox reaction and serves as an important substrate of sirtuins (Sirts) which are involved in maintaining mitochondrial homeostasis [22]. Supplementation of NAD^+^ improves mitochondrial function, and reduces the accumulation of damaged mitochondria and ROS generation in aging and Alzheimer’s disease (AD) models [23–25]. Moreover, it has been shown that NAD^+^ precursor nicotinamide riboside could profoundly alleviate the neuroinflammation in AD mouse models [26, 27]. However, whether NAD^+^ could alleviate the neuroinflammation induced by CCH remains unclear. The aim of this study is to investigate the role of NAD^+^ in CCH-related neuroinflammatory damage.

## Materials and methods

### Animals

Male Sprague-Dawley rats, weighing 160-180 g, 6 weeks old, were housed in SPF-barrier environment under standard conditions of temperature (20-25 °C), humidity (60%), and light (12:12 h light/dark cycle). The rats had free access to food and water. Experimental procedures were performed in accordance with the guidelines of the Chinese legislation on the use and care of laboratory animals and the principles outlined in the National Institutes of Health (NIH) Guide for the Care and Use of Laboratory Animals.

### Chronic cerebral hypoperfusion model and drug administration

The rats were randomly divided into 3 groups (*n* = 15 in each group): (1) sham-operated (sham), (2) bilateral common carotid artery occlusion (BCCAO)-operated (CCH), (3) BCCAO + 250 μg/g/day NAD^+^ (Kaifeng Knature pharmaceutical Co., Ltd., China) for 8 weeks (NAD^+^) [25]. The rats were anesthetized intraperitoneally with sodium pentobarbital (50 mg/kg), and BCCAO operation was used to construct the CCH model. An incision about 1 cm in the middle of the neck was made, and the subcutaneous tissue was bluntly separated. Then, the common carotid artery and vagus nerve on both sides were carefully separated, and the common carotid arteries were cut off after ligation with 4-0 silk suture. Before the incision was sutured, the wound was treated with penicillin. Sham-operated animals received the same procedure without being ligated and cut off the carotid artery.

### Laser speckle contrast imaging

Laser speckle contrast imaging (LSCI) is a technique based on speckle contrast analysis that provides an index of vascular blood flow [28, 29]. A 0.8-mm diameter drill was used to thin the skull and polish the area from the coronal line where the front chimney is located, and then to the coronal line where the apex of the herringbone is located. And then, a 1-mm diameter drill was used to polish the surface of the thinned skull evenly and smoothly, until the blood vessels on the dura are clearly visible. The polished area (5 mm × 5 mm) was marked as the region of interest. The rat was then moved to the laser probe to monitor the cerebral blood flow. The monitoring time of each rat was 5 min. Changes in cerebral blood flow (CBF) before and after bilateral common carotid artery occlusion were measured by using a perfusion speckle imager (Perimed, Stockholm, Sweden).

### Morris water maze tests

In the 8th week after surgery, spatial learning and memory were assessed using a Morris water maze (MWM). The MWM was composed with a circular water pool with a height of 50 cm, a diameter of 180 cm, a water depth of 30 cm. The water temperature of MWM was maintained at 22 ± 1°C. The pool was divided evenly into 4 quadrants where a circular platform with a diameter of 10 cm was placed in the middle of the 2nd quadrant, and the platform is submerged 2 cm below the water surface. There were ample surrounding visual cues around the pool as reference objects. Curtains were mounted around the maze to obscure distal cues. Then tracking systems (View Point, USA) were used for the MWM. Spatial acquisition consisted of four times a day for 5 days. For the training trail, the animal was placed facing the pool wall in the desired quadrant of MWM. The rat was gently released (not dropped) into the water. After the release of animal, the computer automatically started the tracking program. There was 1 min for the rat to search platform. If the rat reached the platform, it was permitted to stay on it for 20 s. If it failed to find the platform within 1 min, the rat was guided to the platform and allowed to stay there for 20 s. In every test, the latency time of reaching the platform was recorded as the escape latency. After 5 days of training, the platform was removed. For the probe trail, the rat was placed in the water facing the pool wall from the opposite of the original platform position. The rat was allowed to navigate freely in water for 60 s. The time spent in the exact quadrant, the number of crossing the location of the platform, and the average swimming speed were recorded.

### Immunostaining

After the MWM test, the brain of the rat was dissected into two hemispheres, one of which was serially embedded in paraffin and cut into 5-μm thick coronal slices for hematoxylin and eosin (HE) staining, while the other hemisphere was processed with optimal cutting temperature compound (OCT) for immunofluorescence. For HE staining analysis, the brain sections were dewaxed, rehydrated, and stained with hematoxylin and eosin according to the manufacturer’s instructions. For immunofluorescent staining, the sections were labeled by the antibody of ionized calcium-binding adapter molecule 1 (Iba1). Briefly, the sections were blocked by PBS containing 3% donkey serum and 0.3% Triton X-100 for 1 h and then incubated with primary Iba1 antibody (1:500, Abcam) overnight at 4 °C. After that, the sections were rinsed, and the Iba1-labeled sections were incubated with secondary antibody (Alexa Fluor 594®, 1:500, CST) for 1 h at room temperature. Finally, the sections were rinsed with PBS and then counterstained with 4′,6-diamidino-2-phenylindole (DAPI). The staining was monitored under a microscope (IX53, Olympus, Tokyo, Japan). Neuronal cells and Iba-1 labeled microglia were quantified in five coronal sections at 100 μm intervals in each rat.

### Dihydroethidium staining

ROS were determined by dihydroethidium (DHE) staining as described previously [30, 31]. Briefly, 10 μm thick frozen brain sections were incubated with 10 mmol/L DHE (Sigma-Aldrich, St. Louis, MO, USA) at 37 °C for 30 min in a humidified chamber without light exposure. The staining was monitored under a microscope (IX53, Olympus, Tokyo, Japan). For the quantitative analysis, the average score was calculated from five randomly selected areas by the Image J software (NIH, USA).

### Transmission electron microscopy (TEM)

Hippocampal tissue was fixed with electron microscopy fixative and preserved at 4°C. Then, the tissue was washed by 0.1 M phosphate-buffered saline (PBS, pH7.4), and fixed with 1% OsO_4_ in 0.1 M PBS (pH 7.4) for 2 h at room temperature. The tissue was then dehydrated in ethanol and embedded in resin. The resin embedded tissue was allowed to polymerize in 65 °C oven for 48 h, and then cut into 60-80 nm thin sections, which were fished out onto the 150 meshes cuprum grids with formvar film. And then, the cuprum grids were stained by uranium acetate-saturated alcohol solution and examined by transmission electron microscopy (HT7800, Hitachi, Tokyo, Japan).

### Transcriptome sequencing analysis

RNA-seq was analyzed by Oebiotech (Shanghai, China). Rat hippocampal RNA (4 rats per group) was extracted to construct cDNA library and perform RNA-seq. Corrected *p* value of 0.05 and log2 (fold change) of 1 were set as the threshold for significant differential expressed genes (DEGs). The DEGs of each group were subjected to the Gene Ontology (GO) and Kyoto Encyclopedia of Genes and Genomes (KEGG) pathway enrichment functional analysis.

### Western blot analysis

Samples of hippocampal tissue or cultured cells were lysed on ice in RIPA buffer containing protease and phosphatase inhibitors (Sigma-Aldrich). Protein concentration was determined by a BCA kit (Sigma-Aldrich). Protein samples (30 μg/sample) were separated by sodium dodecyl sulfate polyacrylamide gel electrophoresis and transferred to polyvinylidene fluoride membranes. The membranes were blocked with 5% fat-free milk and incubated overnight with primary antibodies in TBS-T at 4 °C. Primary antibodies against the following proteins were used in the present study: Sirt1 (1:1000, Abcam), PGC-1α (1:1000, Abcam), and β-actin (1:5000, Abcam). And then, the membranes were washed in TBST, and subsequently incubated with the relevant secondary antibodies for 1 h at room temperature. Immunoreactivities were detected by chemiluminescence reagent (Thermo Scientific) and quantified by a chemiluminescence imaging analyzer (Image Quant LAS 4000, GE, USA). Images were analyzed by the Image J software (NIH, USA).

### qRT-PCR

The total RNA of cultured cells or hippocampal tissue was extracted by EZ-press RNA Purification Kit (EZBioscience, USA). The total RNA was reversed by using Reverse Transcription Master Mix (EZBioscience, USA) at 42 °C for 15 min, 92 °C for 15 s. The SYBR Green qPCR Master Mix (EZBioscience, USA) was used to perform qRT-PCR following the protocol: denaturation (5 min, 95 °C) and 40 amplification cycles (10 s at 95 °C, and 30 s at 60 °C) by using the Strata Gene Mx3000p (Agilent Technologies, Inc., Santa Clara, CA). Interested genes were normalized to β-actin and the fold change was compared relative to the control sample. The sequences of primers are listed in Table 1.

**Table 1:** Primers for qRT-PCR

Gene	Sense (5′-3′)	Anti-sense (5′-3′)
r-IL-1β	TGACCTGTTCTTTGAGGCTGAC	CATCATCCCACGAGTCACAGAG
r-IL-6	CAGCGATGATGCACTGTCAGA	GGAGAGCATTGGAAGTTGGGG
r-TNF-α	GCCACCACGCTCTTCTGTCTA	CGCTTGGTGGTTTGCTACGA
r-iNOS	CAGATCCCGAAACGCTACACT	TGAGTTGAACAAGGAGGGTGGT
r-COX-2	TCAGCCATGCAGCAAATCCTT	TCCAGTCCGGGTACAGTCACA
r-Actin	CGTTGACATCCGTAAAGACCTC	TAGGAGCCAGGGCAGTAATCT
m-IL-1β	TTTGAAGTTGACGGACCCCAA	CACAGCTTCTCCACAGCCACA
m-IL-6	TTCTTGGGACTGATGCTGGTG	CACAACTCTTTTCTCATTTCCACA
m-TNF-α	ATGTCTCAGCCTCTTCTCATTC	GCTTGTCACTCGAATTTTGAGA
m-iNOS	GGGCTGTCACGGAGATCAATG	GCCCGGTACTCATTCTGCATG
m-COX-2	GGGCCATGGAGTGGACTTAAA	TGCAGGTTCTCAGGGATGTG
m-Actin	CTGAGAGGGAAATCGTGCGT	CCACAGGATTCCATACCCAAGA

*r* rat, *m* mouse

### Cell culture and Sirt1 overexpression

The BV2 immortalized mouse microglial cell line purchased from ScienCell (CA, USA) was cultured in DMEM supplemented with 10% FBS, 1% penicillin/streptomycin at 37°C with 5% CO_2_ atmosphere. Stably transfected BV2 cells overexpressing Sirt1 were generated using the overexpression plasmid vector GTP-C-3XFlag. Briefly, Sirt1 (NCBI Gene ID: 93759) was amplified using the following primers: forward primer, 5′-TAGAGCTAGCGAATTCATGGCGGACGAGGTGGCG-3′; reverse primer, 5′- CTTTGTAGTCGGATCCTGATTTGTCTGATGGATAGTTTACA-3′ (Zorin, Shanghai). The amplified sequences were inserted into GTP-C-3XFlag according to the manufacturer’s instructions (Zorin, Shanghai). The constructed plasmid was added to 293T cells. The supernatant containing the virus was collected at 24 h, 48 h, and 72 h. Then, the virus supernatant was used to infect the BV2 cells, and the fluorescence expression in the cells was observed at 24 h and 48 h. To obtain stable transfected lines, the cells were selected with 2.5 μg/ml puromycin. Finally, western blot analysis was used to assess the relative expression level of Sirt1 in transfected BV2 cells.

### Cell stimulation

BV2 cells were assigned into 5 groups: (1) Control: Cells cultured in normal medium without any treatment; (2) Hyp: Cells challenged by hypoxia; (3) Hyp + NC: Cells transfected with Sirt1 negative control sequence and challenged by hypoxia; (4) Hyp + NAD^+^: Cells treated with NAD^+^ and challenged by hypoxia; (5) Hyp + Sirt1: Cells transfected with Sirt1 sequence and challenged by hypoxia. For the hypoxia condition, according to our previous study [32], the oxygen concentration was kept at 3% for 24 h. For the assessment of cell viability, BV2 cells were seeded in 96-well culture plates. After reaching 60% confluence, different concentrations (0 mM, 1 mM, 5 mM, and 10 mM) of NAD^+^ were added to the cells to test the cytotoxic effects. After NAD^+^ exposure, CCK-8 solution (10 μL) was added to each well for 1 h at 37 °C and the absorbance at 450 nm was read on a microplate reader (Thermo Scientific, USA).

### Assays for intracellular ROS and mitochondrial membrane potential

Cells were prepared following the manufacturer’s manual (Beyotime, Shanghai). Briefly, DCFH-DA was diluted with serum-free culture medium at the ratio of 1:1000 to a final concentration of 10 μmol/L. JC-1 dyeing solution was diluted with ultrapure water. The collected cells were loaded with DCFH-DA or JC-1 dyeing solution at 37°C in a humidified atmosphere of 95% air and 5% CO_2_. After 20 min of incubation, the extracellular DCFH-DA and JC-1 were washed away with serum-free culture medium or phosphate buffer saline. Samples were then assessed by flow cytometry (FACS Calibur running CellQuest Pro; Becton Dickinson, UK) or microscope (DM6 B, Leica, Wetzlar, Germany).

### Statistical analysis

The data in our study are expressed as mean ± standard error of the mean (SEM) from three independent experiments, each performed in triplicate. The differences among groups were performed using one-way analysis of variance (ANOVA) followed by Tukey’s post hoc test. For the hidden platform training task of the MWM test, the escape latency was analyzed by two-way repeated-measures ANOVA followed by Tukey’s post hoc test. Values of *P* < 0.05 indicated that the differences were statistically significant. Statistical data was analyzed with the GraphPad Prism 8 software.

## Results

### NAD^+^ treatment mitigated impairment of learning and memory in CCH rats

**Fig. 1:**
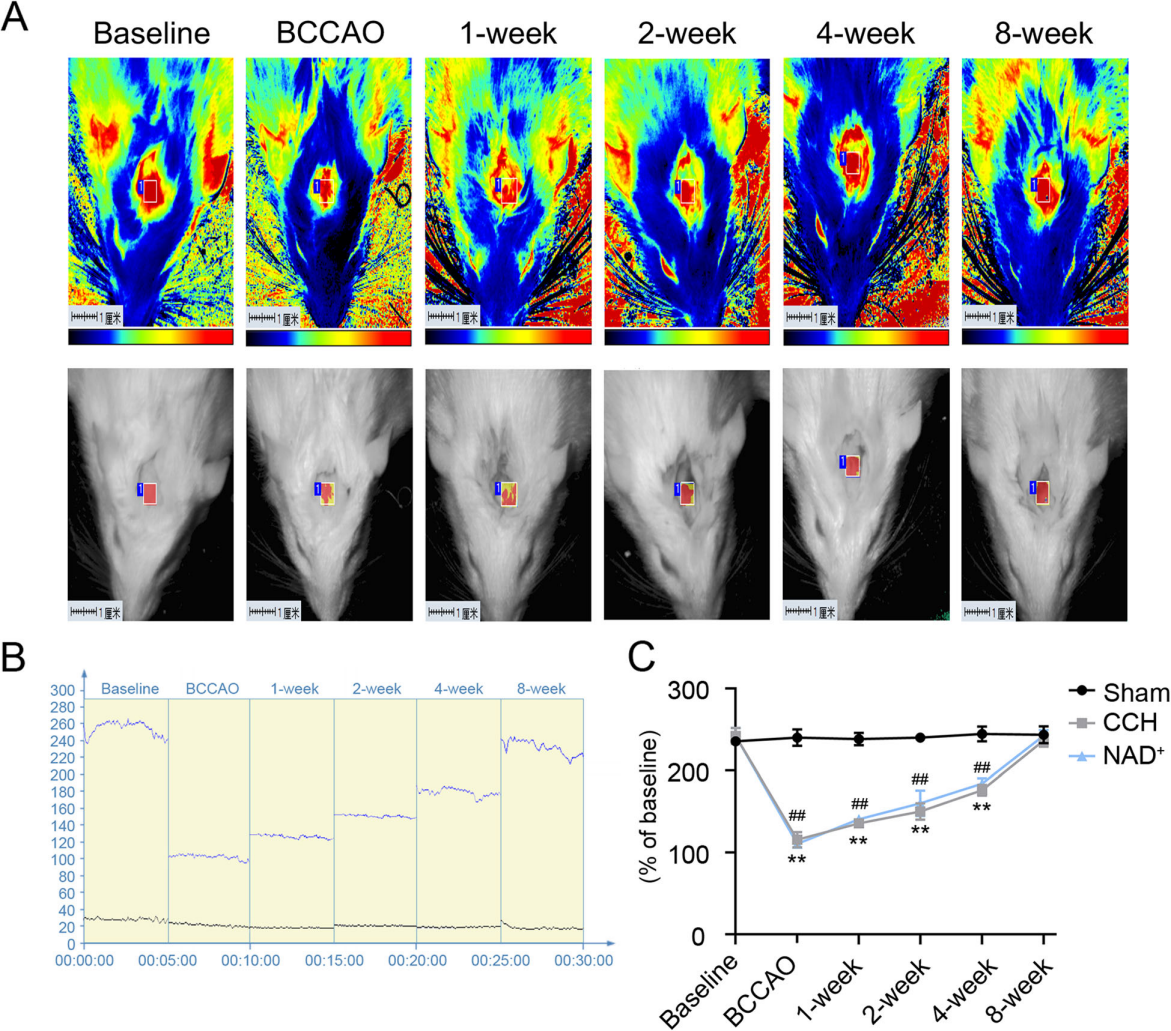
The CCH model was established in rats. **A** Representative contrast and photographic images of cerebral perfusion. **B** CBF absolute values at the following time points: baseline, immediately after BCCAO, and 1, 2, 4, and 8 weeks after BCCAO. Baseline refers to cerebral perfusion before occlusion. **C** Quantification of relative CBF in ROI (% of baseline for each animal). Values are expressed as mean ± SEM (*n* = 3). **P < 0.01, CCH vs. sham; ^##^*P* < 0.01, NAD^+^ vs. sham

**Fig. 2:**
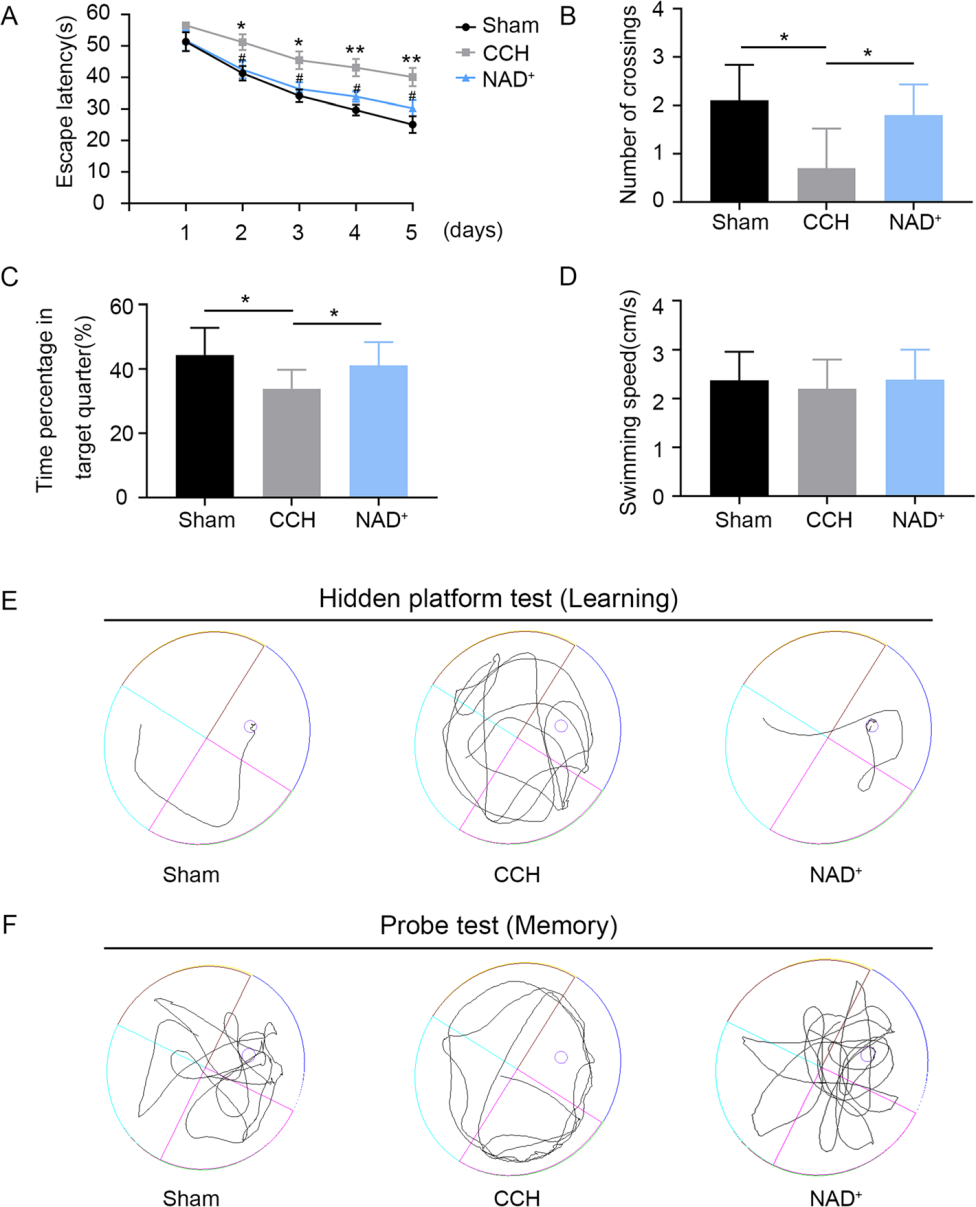
NAD^+^ treatment mitigated CCH-induced learning and memory impairment. **A** The escape latency of rats in the training trials of hidden platform task. **B** The number of crossing the platform in the probe trial. **C** Relative time spent in the target quadrant in the probe trial. **D** Swimming speed in the probe trial. Values are expressed as mean ± SEM (*n* = 10). **E** The representative search traces of  rats in hidden platform test. **F** The representative search traces of rats in probe text. *P < 0.05; **P < 0.01 CCH vs. sham; ^#^*P* < 0.05; NAD^+^ vs. CCH

The BCCAO model is a classic animal model that mimics CCH. After the BCCAO operation, the CBF of rats were monitored for 8 weeks by LSCI. The CBF tests (Fig. 1 A-C) showed that the CBF decreased immediately after the operation and then recovered slowly. At the 8th week of the surgery, the CBF was close to the baseline. The results of CBF test were consistent with previous research [32] showing that the CCH model was successfully constructed. Moreover, the MWM test is the most widely-employed laboratory behavioral test for the assessment of cognitive deficits in rodents [33]. We performed the MWM test 8 weeks after BCCAO/sham operation. In the hidden platform task, the escape latency of CCH group was significantly prolonged compared with the sham group, and the deterioration of the CCH rats was protected by administration of NAD^+^ (Fig. 2A and E). In the probe trial, the number of platform crossing and the time spent in the target quadrant of the CCH group were decreased compared with the sham group. However, treatment with NAD^+^ improved these cognitive deficits in CCH rats (Fig. 2B, C, F). In addition, there was no significant difference in swimming speed between the tested groups (Fig. 2D). These results showed that NAD^+^ treatment ameliorated the impairment of learning and memory induced by CCH.

### NAD^+^ reduced neuronal death and microglial activation in CCH rats

**Fig. 3:**
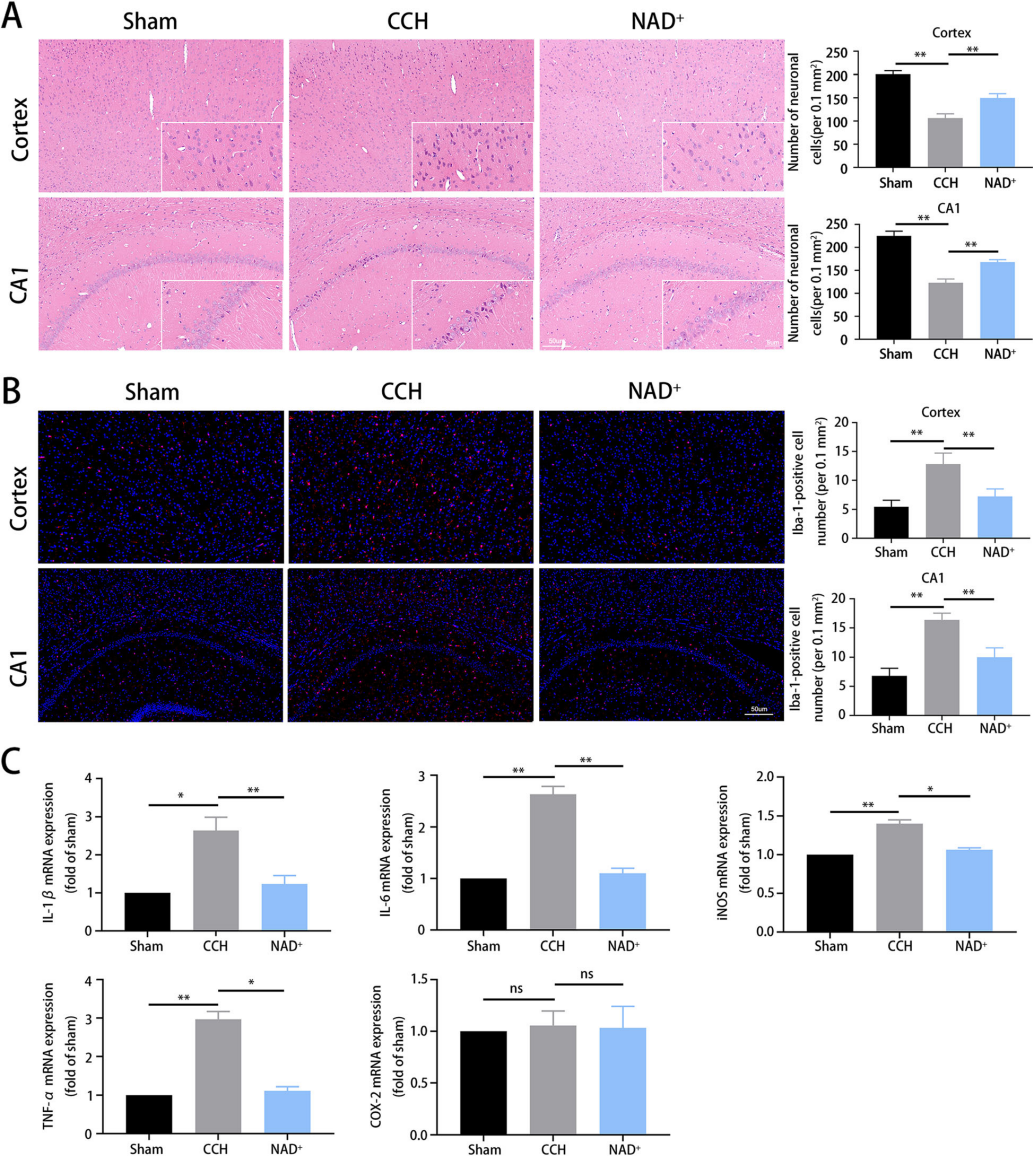
NAD^+^ reduced CCH-induced neuronal death and microglial activation. **A** HE staining of cortex and hippocampus CA1 at different magnifications (scale bar = 50 μm or 5 μm). **B** Representative immunofluorescent staining for Iba1-labeled microglia (red). Nuclei were stained with DAPI (blue). Scale bar = 50 μm. **C** The mRNA expression of pro-inflammatory cytokines (IL-1β, IL-6, iNOS, TNF-α, and COX2) was detected by qRT-PCR. *P < 0.05; **P < 0.01 sham and NAD^+^ vs. CCH

Hippocampal (CA1) and cortical neuronal damage is the main pathological change induced by CCH which leads to cognitive impairment [34]. HE staining of cortex and hippocampus showed that the neuronal death was increased in CCH compared with the sham group. As shown in Fig. 3A, the number of neurons in the CCH group was reduced and the morphology was abnormal as the cells were shrunk, and the nucleus was deeply stained. The abnormality was mitigated by NAD^+^ treatment. Neuroinflammation plays an important role in the etiology of CCH [4]. In order to detect the regulatory effect of NAD^+^ on neuroinflammation, immunofluorescent staining was performed to examine the microglia on hippocampal sections. It was found that the number of Iba1-labeled microglia was significantly increased in CCH group compared with the sham group, and NAD^+^ treatment reduced the increase (Fig. 3B). To further detect the level of pro-inflammatory factors, qRT-PCR was performed to assess the expression of IL-1β, IL-6, TNF-α, COX-2, and iNOS in hippocampal tissue. The results (Fig. 3C) showed that expression of IL-1β, IL-6, TNF-α, and iNOS in the CCH group was higher than that in the sham group but there was no significant difference in COX-2, while NAD^+^ administration reversed the increase. These results demonstrated that NAD^+^ treatment alleviated CCH-induced neuronal death, microglial activation and pro-inflammatory factor expressions in cerebral cortex and hippocampus.

### NAD^+^ alleviated CCH-induced ROS generation and mitochondrial injury

**Fig. 4:**
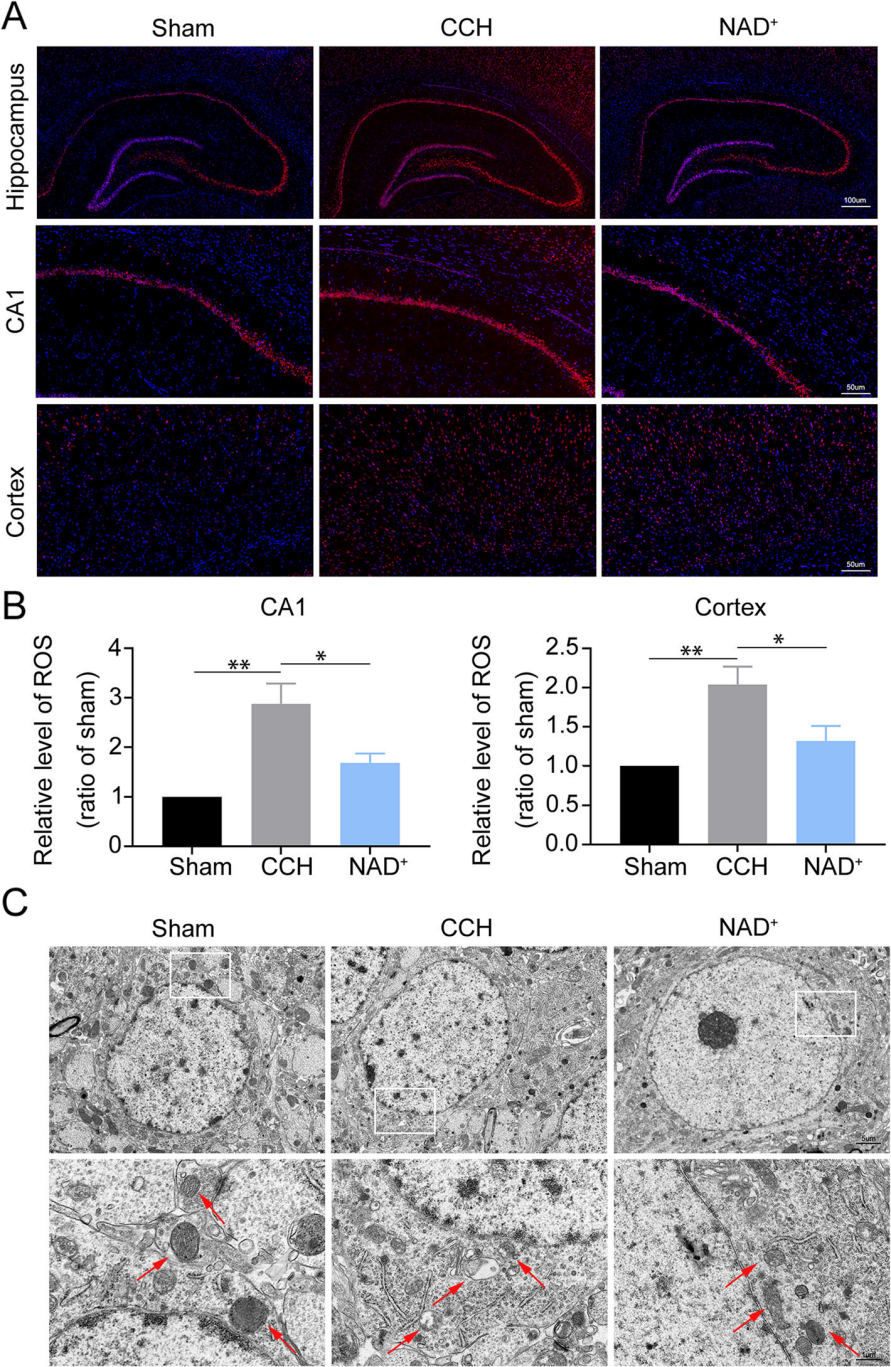
NAD^+^ alleviated ROS generation and mitochondrial injury. **A** Representative DHE fluorescence staining for ROS in the hippocampus and cortex (scale bars = 50 μm). **B** Relative level of ROS (ratio to sham group). Data are expressed as mean ± SEM (*n* = 3). *P < 0.05; **P < 0.01 vs. CCH. **C** Representative TEM micrographs of mitochondria in hippocampal microglia (scale bars = 5 μm or 1 μm)

Studies have shown that excessive generation of intracellular ROS activates microglia and causes secondary cell damage [18, 19]. To explore whether NAD^+^ can reduce neuroinflammation by attenuating ROS production, we evaluated the level of ROS. We found that ROS generation in the cortex and hippocampus of CCH rats was significantly increased compared with the sham group, and the increase was reversed by treatment with NAD^+^ (Fig. 4A and B). Moreover, damaged mitochondria are the main source of intracellular ROS [35]. Therefore, we observed ultrastructural changes of mitochondria in hippocampal microglia by TEM. As shown in Fig. 4C, in the sham group, the mitochondria of microglia had prominent cristae and intact membrane, while in the CCH group, the mitochondria were swelled, and the cristae was broken and moved to the surrounding. This result suggested that damaged mitochondria in microglia may be one of the sources of CCH-induced ROS. Administration of NAD^+^ ameliorated these damages. The above results suggested that NAD^+^ treatment suppressed ROS derived from damaged mitochondria of microglia in cerebral cortex and hippocampus of CCH rats.

### NAD^+^ activated Sirt1/PGC-1α pathway in CCH model

**Fig. 5:**
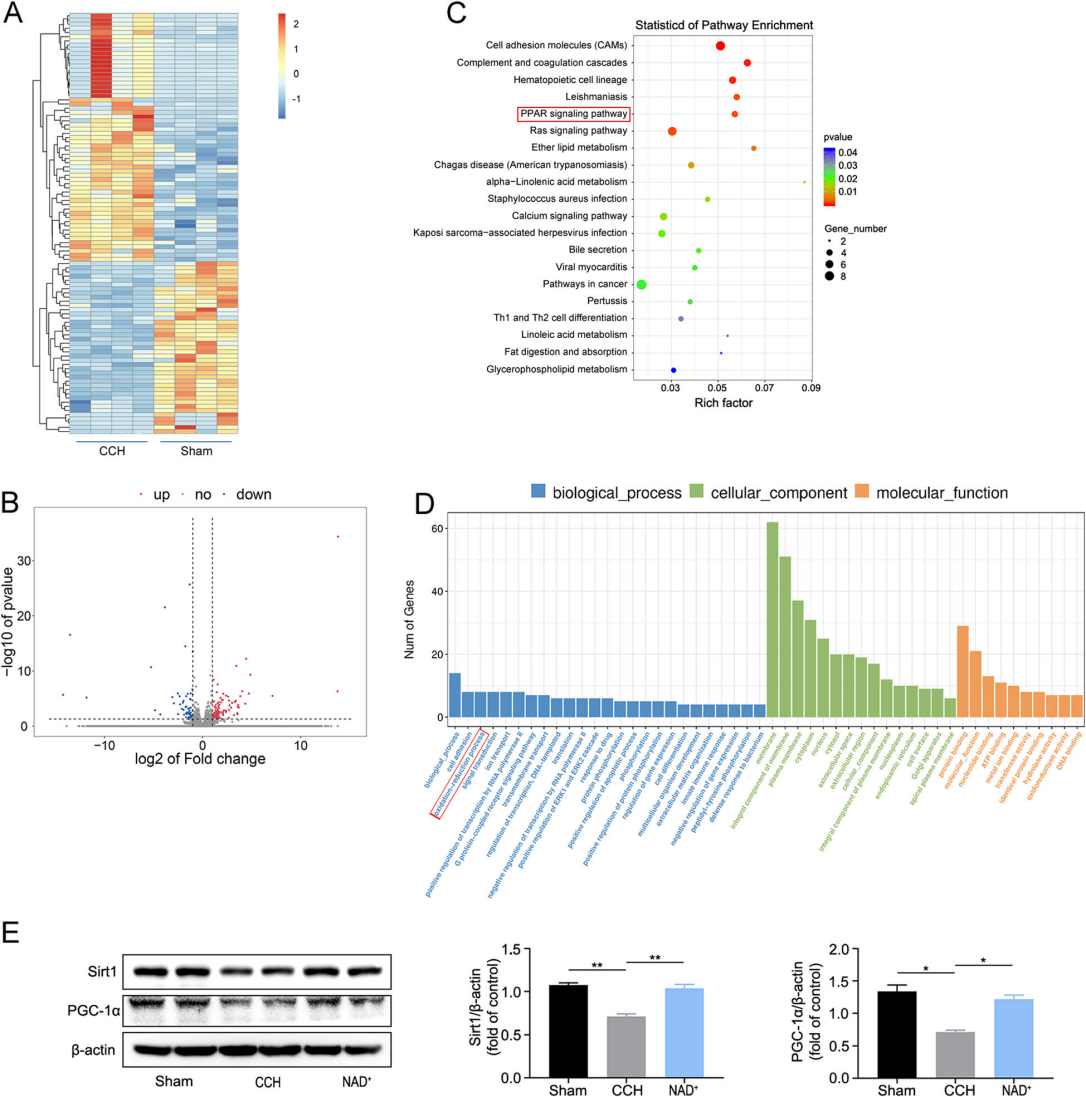
Exploration of potential mechanisms of NAD^+^ effects in CCH model. **A** RNA-seq results showing hierarchical clustering of co-regulated DEGs. **B** Volcano plotting of DEGs. **C** The significantly changed genes that were analyzed by KEGG enrichment. **D** List of enriched GO term on biological process, cellular component, and molecular function. **E** Western blot analysis of Sirt1, PGC-1α in hippocampus. Data are expressed as mean ± SEM (*n* = 3). Data are expressed as mean ± SEM (*n* = 3). *P < 0.05, **P < 0.01, vs. CCH

To explore the mechanism of the CCH-induced damage, RNA-seq analysis was carried out in the hippocampal samples. The differentially expressed genes in CCH rats were screened out as fold change (≥ 2) and *p* value (≤ 0.05) compared with the sham group (Fig. 5A and B). There were 100 genes upregulated and 64 genes downregulated in CCH group. GO analysis is commonly used to comprehensively describe the attributes of genes in organism including biological process, cellular component, and molecular pathway function [36]. As shown in (Fig. 5D), the results of GO analysis revealed significant enrichment in redox activity and signal transduction. KEGG provides a reference knowledge base for linking genomic or transcriptomic content of genes to KEGG reference pathways inferring systemic behaviors of the organism [37]. According to the KEGG pathway enrichment analysis (Fig. 5C), peroxisome proliferator-activated receptor (PPAR) signaling pathway was significantly enriched. To summarize, the functional analysis of DEGs indicated that CCH may cause pathological damage by changing the redox reaction and PPAR pathway.

According to previous reports [38, 39], biological activities of PPARs are associated with the PPAR-γ co-activator-1α (PGC-1α). PGC-1α is a master transcription factor in the regulation of mitochondrial antioxidant and clearance systems, as well as its biogenesis [40]. Furthermore, PGC-1α is regulated by the upstream transcription factor Sirt1. Therefore, in order to further verify the results of RNA-seq, we extracted proteins from the hippocampal tissue and performed western blot analysis. It was found that the expression of Sirt1 and PGC-1α in the CCH group was reduced significantly compared with the sham group, while they were increased after the administration of NAD^+^ (Fig. 5E). The results implied that NAD^+^ reduced the mitochondria-derived ROS and finally suppressed the neuroinflammation caused by ROS partially through regulating Sirt1/PGC-1α pathway.

### NAD^+^ and overexpression of Sirt1 reduced the generation of pro-inflammatory cytokines in hypoxia-challenged BV2 microglia

**Fig. 6:**
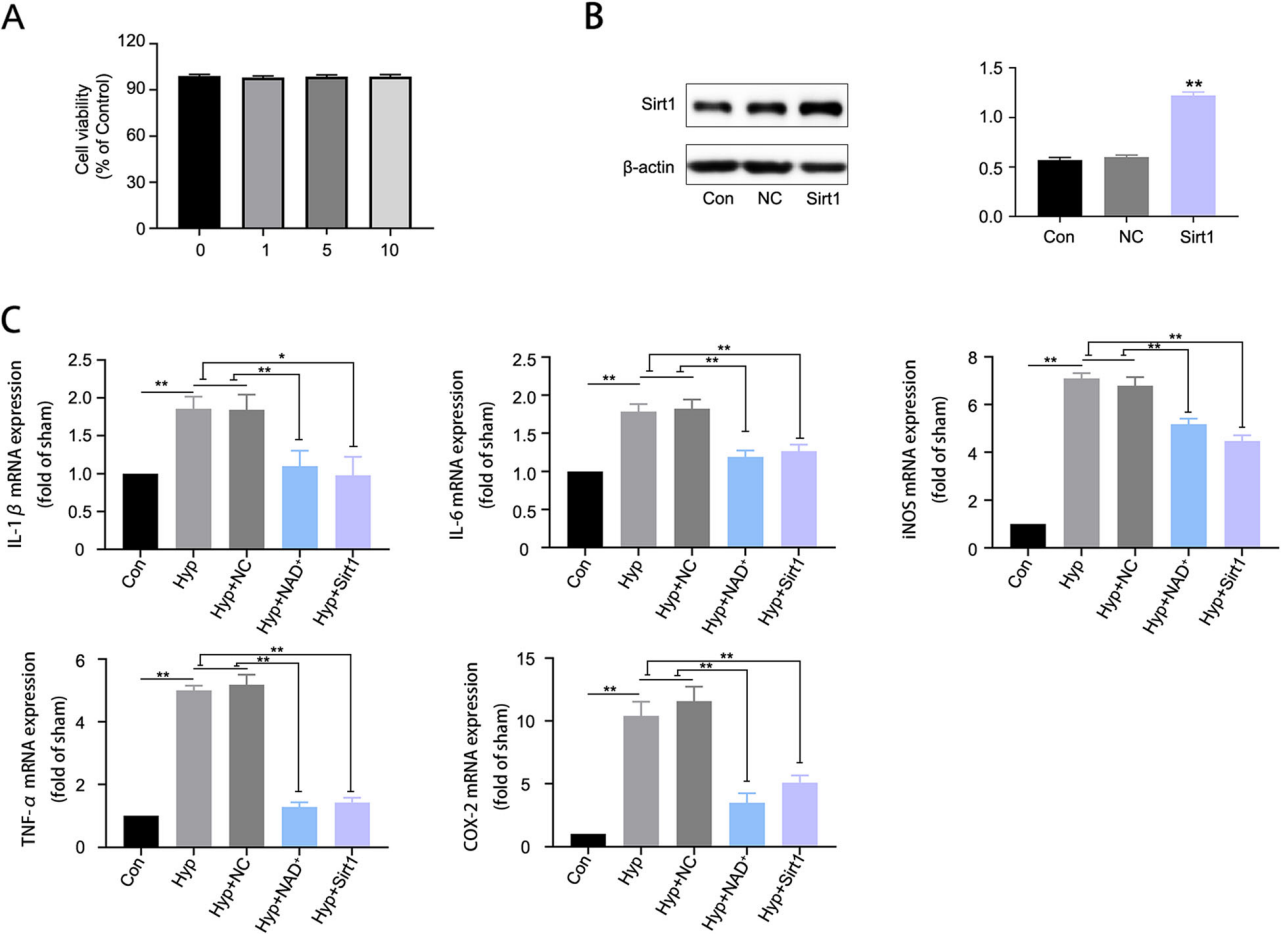
NAD^+^ and overexpression of Sirt1 reduced the generation of pro-inflammatory cytokines in hypoxia-challenged microglia. **A** Cell viability results of BV2 microglial cells exposed to 0 mM, 1 mM, 5 mM, and 10 mM NAD^+^ for 24 h. **B** Western blot for Sirt1 in BV2 microglia transfected with Sirt1 lentiviral particles. Data are expressed as mean ± SEM (*n* = 3), **P < 0.01 vs. control. **C** The mRNA expression of pro-inflammatory markers (IL-1β, IL-6, iNOS, TNF-α, and COX2) was assessed by qRT-PCR. Data are expressed as mean ± SEM (*n* = 3). *P < 0.05, **P < 0.01 vs. Hyp and Hyp+NC

In order to further demonstrate the effects of NAD^+^ in vitro, hypoxia-challenged BV2 microglia were treated with NAD^+^ or overexpressed with Sirt1. We found NAD^+^ of different concentrations had no significant effect on cell viability (Fig. 6A), and we chose NAD^+^ concentration of 5 mM for NAD^+^ experiment in hypoxia-challenged microglia [41]. Sirt1 overexpression was confirmed by western blot analysis (Fig. 6B). To verify the effects of NAD^+^ on neuroinflammation induced by hypoxia in BV2 microglia, we examined the expression of pro-inflammatory markers IL-1β, IL-6, TNF-α, COX-2, and iNOS. The results (Fig. 6C) showed that compared with control group, hypoxia increased the generation of pro-inflammatory cytokines, while NAD^+^ and overexpression of Sirt1 induced a prominent reduction. These data were basically consistent with the in vivo results. The above results demonstrated that NAD^+^ and overexpression of Sirt1 attenuated the generation of pro-inflammatory cytokines in hypoxia-challenged microglia.

### NAD^+^ alleviated hypoxia-induced mitochondrial injury by promoting the expression of Sirt1/PGC-1α in BV2 microglia

**Fig. 7:**
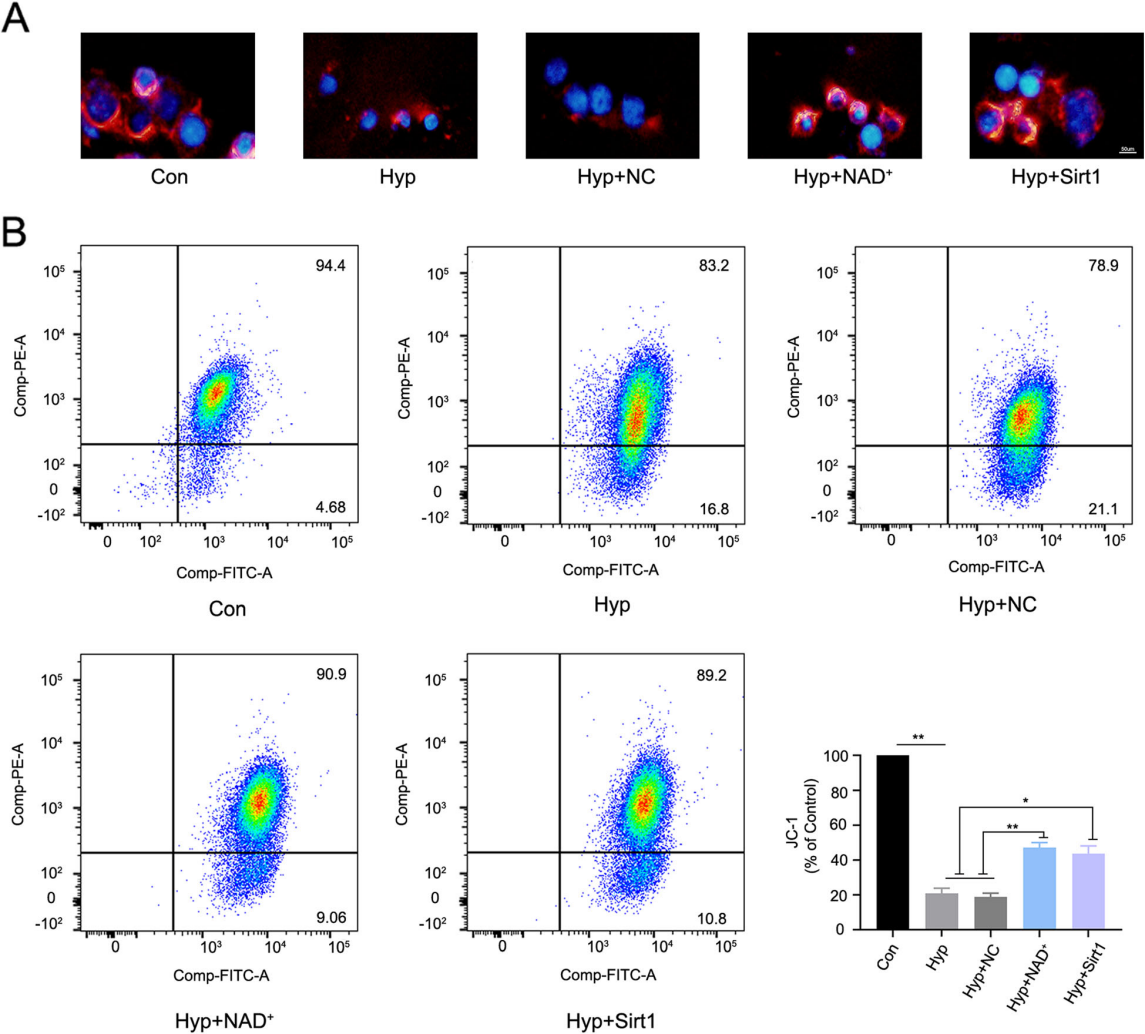
NAD^+^ and overexpression of Sirt1 attenuated mitochondrial injury in hypoxia-challenged BV2 microglia. **A** Representative image of BV2 microglia loaded with the JC-1 (red). **B** Flow cytometry analysis of JC-1 in BV2 microglia. Data are expressed as mean ± SEM (*n* = 3), *P < 0.05, **P < 0.01 vs. Hyp and Hyp + NC

**Fig. 8:**
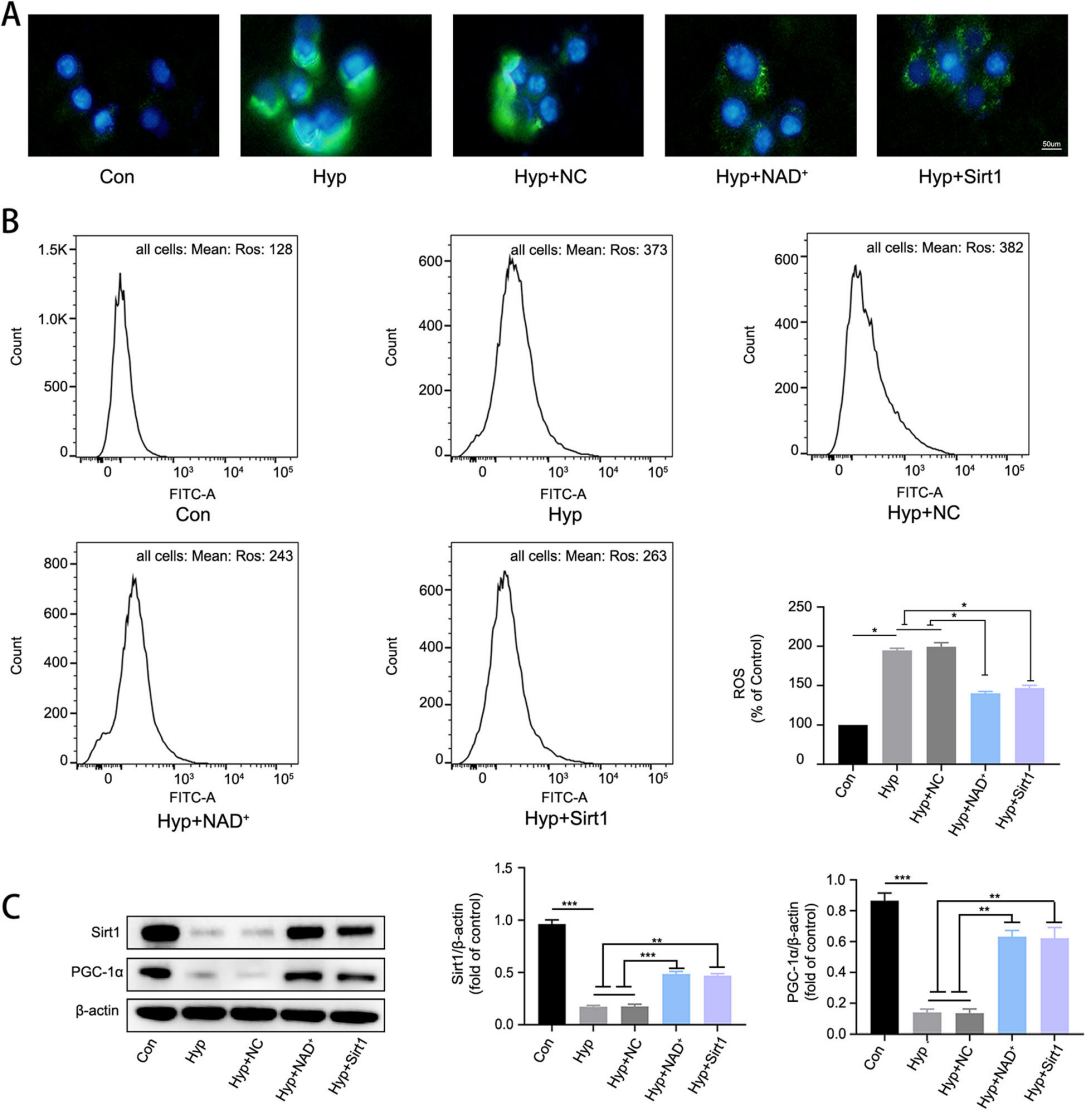
NAD^+^ alleviated hypoxia-induced ROS production by promoting the expression of Sirt1/PGC-1α. **A** Representative image of microglia loaded with ROS indicator DCF-DA (green). **B** Flow cytometry analysis of ROS production in BV2 microglia. Data are expressed as mean ± SEM (*n* = 3), *P < 0.05, **P < 0.01 vs. Hyp and Hyp + NC. **C** Western blot analysis of Sirt1 and PGC-1α. Data are expressed as mean ± SEM (*n* = 3). *P < 0.05, **P < 0.01 vs. Hyp and Hyp + NC

To test the protective effects of NAD^+^ in vitro, we performed fluorescence microscopy and flow cytometry assays to investigate mitochondrial membrane potential and ROS. As shown in Fig. 7A and B, the mitochondrial membrane potential of BV2 microglia was decreased by hypoxia challenge, while NAD^+^ administration or Sirt1 overexpression rescued the decrease. Furthermore, we found that hypoxia increased the generation of ROS in BV2 microglia, while administration of NAD^+^ and overexpression of Sirt1 reduced ROS production (Fig. 8A and B). To verify whether NAD^+^ acts through Sirt1/PGC-1α pathway, we performed western blot analysis of Sirt1/PGC-1α in BV2 microglia. We found that hypoxia induced a decline in the levels of Sirt1/PGC-1α, and such a decline was significantly attenuated by NAD^+^ treatment or overexpression of Sirt1 (Fig. 8C). The above results indicated that NAD^+^ may alleviate hypoxia-induced mitochondrial injury and decrease ROS production by promoting the expression of Sirt1/PGC-1α.

## Discussion

The present study shows that CCH could induce cognitive impairment through intracellular ROS generation, mitochondrial injury, and neuroinflammation; and NAD^+^ treatment could rescue the damage of mitochondria, decrease ROS production, reduce neuroinflammatory response, and ultimately improve the cognitive dysfunction in CCH models in vivo and in vitro. Such neuroprotective effects are at least in part associated with NAD^+^ induced Sirt1/PGC-1α pathway activation in microglia.

Vascular cognitive impairment (VCI) can occur as a result of persistent cerebral hypoperfusion when CBF is globally or focally decreased in patients. The CCH model mimics global chronic cerebral hypoperfusion in aging and dementia in human [34]. It has been demonstrated that the CBF in cortex and hippocampus significantly decreased after CCH operation, and gradually recovered to normal level at 8 weeks after CCH induction [3]. In our study, we used LSCI to detect the changes of CBF before and after the CCH operation in rats, and the results of CBF test were consistent with the previous findings [3, 32]. According to previous report [25], the toxic effects of NAD^+^ and its precursors have been extensively studied. The dosage used is in the range of 40 mg/kg/day to 400 mg/kg/day, which has been demonstrated as not toxic. We selected the 250 mg/kg/day dose that has also been tested in another article [42]. Thus, we did not set a separate NAD^+^ administration group in our study. For the in vitro model in BV2 microglia, we found that 3% O_2_ for 24 h caused damage to microglia, thus verifying the results of our previous study [32]. Collectively, the present study had successfully established in vitro and in vitro models of CCH.

Hippocampal (CA1) and cortical neuronal damage are the main pathological changes induced by CCH which leads to cognitive impairment [34]. We found that NAD^+^ treatment significantly ameliorated CCH-induced neuronal damage in the cortex and hippocampal CA1 area, and improved cognitive impairment. Neuroinflammation has a crucial role in the pathogenesis of cerebrovascular and neurodegenerative diseases [43, 44], and microglia play key roles therein. Activated microglia release pro-inflammatory factors such as IL-1β, IL-6, TNF-α, COX-2, and iNOS, which further promote neuronal degeneration in AD and CCH model [34, 45]. Therefore, regulating microglia and reducing neuroinflammation have been considered as a promising strategy to treat dementia. Our results showed that CCH could induce the activation of microglia in vivo, and NAD^+^ treatment significantly reduced the number of activated microglia. Furthermore, the results of qRT-PCR demonstrated NAD^+^ treatment significantly reduced the increased levels of pro-inflammatory factors including IL-1β, IL-6, TNF-α, and iNOS in hippocampus of CCH rats. The in vitro results also indicated that NAD^+^ alleviated hypoxia-induced production of pro-inflammatory factors in microglia. These results demonstrate that NAD^+^ can alleviate neuroinflammation, the neuronal damage, and cognitive impairment in CCH model. Contemporary studies demonstrate similar results that NAD^+^ can attenuate pro-inflammatory activation in heart failure [46], and ameliorate experimental acute kidney injury triggered by systemic inflammation [47].

ROS plays an important role in inflammatory disorders [48]. Chronic or prolonged ROS production is considered a key contributor to inflammatory diseases, and the role of ROS in inflammation has been vigorously investigated in various disease models [49–51]. Previous studies have shown CCH could induce ROS accumulation [52, 53]. Therefore, we explored whether NAD^+^ could reduce neuroinflammation by attenuating ROS production. Our results showed that ROS levels in brain tissue of CCH rats were increased, while NAD^+^ administration significantly reduced ROS levels. These results were consistent with previous studies of NAD^+^ effects on ROS metabolisms in models of brain ischemia [54, 55]. Mitochondria are key regulators of cellular energy metabolism and redox balance, and damaged mitochondria are important sources of intracellular ROS [35, 56]. Dysfunction of mitochondria affects cell metabolism and results in increased generation of ROS, which in turn triggers inflammation [57, 58]. By TEM study, we showed that CCH induced mitochondrial swelling and cristae abnormality in microglia, implying CCH-induced ROS might originate from damaged mitochondria. On the other hand, NAD^+^ treatment rescued the mitochondrial damage and reduced ROS production in CCH rats. In vitro study also indicated that NAD^+^ alleviated hypoxia-induced decrease in mitochondrial membrane potential and reduced the production of ROS and pro-inflammation cytokines. As for the pro-inflammation cytokines COX-2, the RNA expression level is consistent with our previous experimental results [32]. There is no obvious difference in the RNA expression level of COX-2 in the animal model, but there is a difference in the cell model. We attribute this difference to the inability of the cell model to fully simulate the in vivo state for this biomarker. Thus, our findings reveal the treatment effects of NAD^+^ against CCH-induced neuroinflammation are at least partially through protecting mitochondria function and inhibiting ROS production.

To further explore the mechanism of NAD^+^ in a transcriptome level, we performed RNA-seq analysis of the hippocampal tissue. The results showed that CCH significantly changed the redox reaction process and PPAR pathway. PPARs are nuclear hormone receptors that regulate genes involved in energy metabolism and inflammation [59]. The biological function of PPARs relies on PPAR-γ co-activator1α (PGC-1α) for co-activation [39]. Meanwhile, PGC-1α maintains mitochondrial homeostasis by regulating mitochondrial dynamics and energy and increasing mitochondrial abundance [60, 61]. PGC-1α is a master transcription factor in the regulation of mitochondrial antioxidant and clearance systems, as well as its biogenesis [40]. Mitochondrial biogenesis can be defined as the growth and division of pre-existing mitochondria. Mitochondrial structure and number are not static, rather changes during development and in response to increased energy demands or physiological stimulations [62]. Reduction in mitochondrial function has been found to be associated with many pathologies associated with aging, such as type 2 diabetes and Alzheimer’s disease [63]. The steady state number of mitochondria represents an equilibrium between mitochondrial biogenesis and eventual degradation through the process of mitophagy [64]. The PGC-1 family consists of three members, namely, PGC-1α, PGC-1β, and the PGC-related coactivator (PRC). PGC-1α, in particular, is often cited as a master regulator of this process [65]. PGC-1α co-activates the transcription of nuclear respiratory factors (NRF) 1 and 2, which, in turn, regulate the transcription of mitochondrial transcription factor A (TFAM). TFAM translocates into mitochondrial matrix where mitochondrial DNA (mtDNA) replication and mitochondrial gene expression are stimulated [65]. Therefore, in the model of this experiment, we regard the decrease of damaged mitochondria and the increase of healthy mitochondria as the result of mitochondrial biogenesis promoted by PGC-1α. Besides, the previous study had showed that in the renal tubule, PGC-1α improved mitochondrial quality to rescue acute renal injury induced by genotoxic stressor cisplatin [66]. In diabetic cardiovascular injury, PGC-1α provides cardiovascular protection through decreasing mitochondrial ROS production and dampening inflammation [67]. It has been well established that the upstream transcription factor Sirt1 regulates PGC-1α by increasing its expression and decreasing its acetylation [68]. Moreover, NAD^+^ serves as a substrate of Sirt1 and regulates Sirt1 expression [69]. Sirt1 has been shown to play protective roles in several neurodegenerative diseases including Alzheimer’s, Parkinson’s, and motor neuron diseases, which may relate to its functions in metabolism, stress resistance, and genomic stability [70]. It has been reported that Sirt1 is essential in maintaining normal cognitive function, and Sirt1 knockout mice have impaired hippocampal-dependent memory [71]. Our data showed that CCH decreased the expression of Sirt1 and PGC-1α, while NAD^+^ treatment reversed the decrease in vivo and in vitro. We also showed that Sirt1 overexpression in BV2 cells mimicked NAD^+^ effects in improving mitochondrial damage and reducing neuroinflammation. Thus, the treatment effects of NAD^+^ on CCH models might be mediated by the activation of Sirt1/PGC-1α pathway.

## Conclusions

Collectively, our data revealed that NAD^+^ improved cognitive function and reduced neuroinflammation in CCH models in vivo and vitro, and these treatment effects were associated with mitochondrial protection and ROS inhibition through the activation of Sirt1/PGC-1α pathway. These findings imply NAD^+^ may be a potential novel therapeutic method in CCH-induced cognitive impairment, and targeting Sirt1/PGC-1α pathway may be a promising treatment strategy in VaD.

## Data Availability

All data generated or analyzed during this work are available upon request.

## Abbreviations

VaD: Vascular dementia
CCH: Chronic cerebral hypoperfusion
ROS: Reactive oxygen species
BCCAO: Bilateral common carotid artery occlusion
LSCI: Laser speckle contrast imaging
NAD^+^: Nicotinamide adenine dinucleotide
Sirt1: Sirtuin1
PPAR: Peroxisome proliferator-activated receptor
PGC-1α: PPAR-γ co-activator1α
Hyp: Hypoxia
DEGs: Differential expressed genes
GO: Gene Ontology
KEGG: Kyoto Encyclopedia of Genes and Genomes
VCI: Vascular cognitive impairment
